# Survival and revision causes of hip resurfacing arthroplasty and the Mitch proximal epiphyseal replacement: results from the Danish Hip Arthroplasty Register

**DOI:** 10.1080/17453674.2019.1646201

**Published:** 2019-07-25

**Authors:** Maja Tang-Jensen, Per Kjærsgaard-Andersen, Thomas K Poulsen, Søren Overgaard, Claus Varnum

**Affiliations:** a Department of Orthopaedic Surgery, Vejle Hospital, Vejle;; b Department of Clinical Research, University of Southern Denmark, Odense;; c Department of Orthopaedic Surgery and Traumatology, Odense University Hospital, Denmark

## Abstract

Background and purpose — The Mitch proximal epiphyseal replacement (PER) was developed to preserve proximal femoral bone and minimize femoral neck fracture associated with hip resurfacing arthroplasty (HRA). We studied the survival and risk of revision of HRA compared with cementless metal-on-polyethylene (MoP) total hip arthroplasty (THA) and the survival and risk of revision of the Mitch PER compared with MoP THA.

Patients and methods — Using propensity score, we matched 1,057 HRA to 1,057 MoP THA and 202 Mitch PER to 1,010 MoP THA from the Danish Hip Arthroplasty Register. To estimate the relative risk (RR) of revision, we used regression with the pseudo-value approach and treated death as a competing risk.

Results — The cumulative incidence for any revision of HRA at 10 years’ follow-up was 11% (95% confidence interval [CI] 9.1–13) and 6.4% (CI 5.8–7.0) for MoP THA. The RR of any revision was 1.5 (CI 1.1–2.1) for HRA at 10 years’ follow-up. By excluding the ASR components, the RR of revision at 10 years was 1.2 (CI 0.8–1.7). The cumulative incidence of revision was 9.6% (CI 4.2–18) for Mitch PER and 5.4% (CI 5.1–5.7) for MoP THA at 8 years. The RR of revision was 2.0 (CI 0.9–4.3) for Mitch PER at 8 years’ follow-up.

Interpretation — The HRA had increased risk of revision compared with the MoP THA. When excluding ASR, the HRA group had similar risk of revision compared with MoP THA. The Mitch PER did not have a statistically significant increased risk of revision compared with MoP THA.

Metal-on-metal (MoM) hip resurfacing arthroplasty (HRA) is still used in younger and more active patients in some countries (Marshall et al. 2014). Disadvantages of MoM and HRA include early implant failure with specific designs, especially the ASR Hip System from DePuy (de Steiger et al. 2011), proximal femoral bone resorption, femoral neck fracture (Marshall et al. 2014), and adverse reactions to metal debris (ARMD) with reports of pseudotumors (Pandit et al. 2008, Langton et al. 2011). These factors have led to a clear drop in the use of MoM bearings in general, and since 2012 Danish national guidelines on MoM and HRA have advocated to stop the use of these implants and very few have been inserted since.

The Mitch proximal epiphyseal replacement (PER) (Figure 1) was developed by Finsbury Orthopaedics and first used in 2005. It was developed to solve the problems with femoral neck fractures of HRA and secondary proximal femoral bone resorption as it was designed to preserve patient bone and strengthen the femoral neck. It has gone through extensive computer simulation studies using the finite element method, a powerful tool to investigate the mechanical behavior of structural components. The results showed that the risk of femoral neck fracture was not influenced by the presence of the revised implant and the femoral neck strength increased after implantation from 9% to 49% compared with a previous study (Martelli et al. 2011).

**Figure 1.**
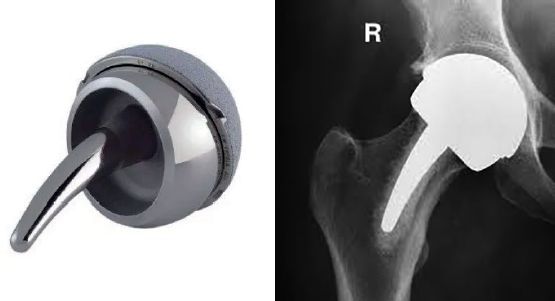
The Mitch proximal epiphyseal replacement (PER).

**Figure 2.**
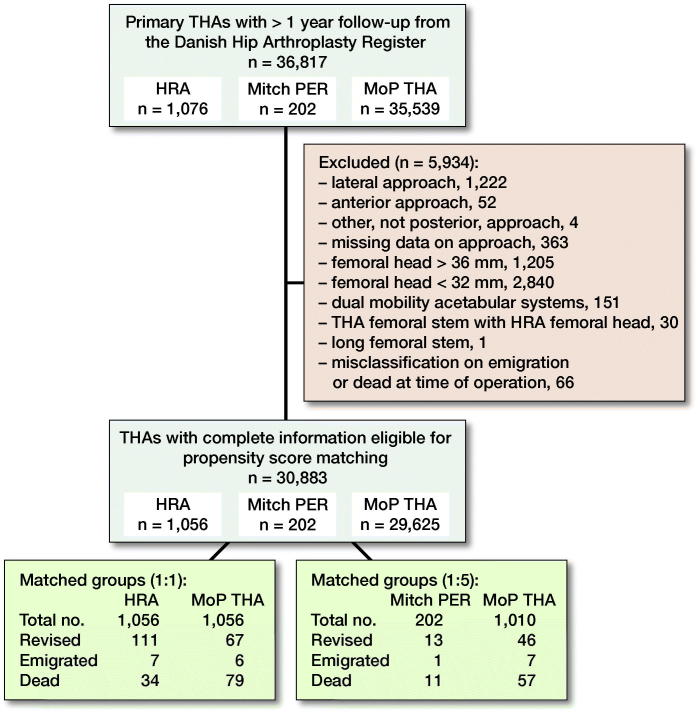
Flow diagram: inclusion of hips with hip resurfacing arthroplasty (HRA), Mitch proximal epiphyseal replacement (PER), or cementless metal-on-polyethylene total hip arthroplasty (MoP THA) in the study population.

To our knowledge, there are no clinical studies of the Mitch PER or any similar prosthesis. Further, there are no nationwide Danish mid-term results on HRA. Therefore, we examined the survival and risk of revision of HRA and Mitch PER compared with a control group of cementless MoP THA based on nationwide data from several registers in Denmark. Further, we studied the revision risk of different designs of HRAs and the causes of revision. 

## Patients and methods

This Danish register-based study with prospectively collected data was based on data from the Danish Hip Arthroplasty Register (DHR), the Civil Registration System (CRS), and the Danish National Patient Registry (DNPR). The population in Denmark is approximately 5.8 million and every citizen is entitled to tax-funded “free” health care.

### Data sources

The DHR is a nationwide population-based clinical database, containing prospectively collected data on primary THA and revisions. The DHR was established in 1995, validated in 2004 (Pedersen et al. 2004) and has almost complete coverage as reporting is compulsory. The completeness for primary THA is 98% and 95% for revisions using the DNPR as a reference (Danish Hip Arthroplasty Registry 2018).

The CRS is an administrative register founded in 1968. It holds information on vital status, sex, date of birth, and residence on all persons residing in Denmark (and Greenland from 1972). Every Dane is given a unique 10-digit identification number at birth that allows unambiguous linkage between all medical databases in Denmark. The CRS is updated daily, is virtually complete with a prevalence of disappeared persons around 0.3%, and is checked systematically for errors (Schmidt et al. 2014). We used data from the CRS to account for censoring due to emigration or death. Missing persons and changed CRS number were treated as emigrated as we have the exact date they went missing or changed CRS number.

Since 1977, the DNPR has collected data on non-psychiatric patient visits from Danish hospitals and from 1978 with nationwide coverage. Diagnosis is classified according to the Danish version of the International Classification of Diseases (ICD) and since 1993 the 10th edition (ICD-10) has been used (Schmidt et al. 2015). We used the DNPR to identify patients with comorbidity and determine the Charlson Comorbidity Index (CCI), based on diagnoses registered in the DNPR. It contains 19 major disease categories and brings the comorbidities down to 1 single numeric score (Thygesen et al. 2011). For each patient at time of surgery, the CCI was classified into 3 groups: low (CCI 0), medium (CCI 1 to 2), high (CCI 3 or more). The coverage and completeness of the 19 Charlson conditions have all been validated and have an overall positive predictive value of 98% (Thygesen et al. 2011).

### Study population (Figure 2)

This study is reported according to the RECORD guidelines.

From the DHR, we identified patients with HRA, Mitch PER, or primary cementless MoP THAs with highly crosslinked polyethylene and minimum 1 year of follow-up (n = 36,817). The first HRAs were followed from 2005 and Mitch PER from 2008 until end of the study period in 2016.

THAs with missing information on approach or approach other than posterior (n = 1,641) were excluded as these approaches are not commonly used. THAs with a femoral head size smaller than 32 mm (n = 1,222) were excluded, because they have a greater risk of dislocation (Kostensalo et al. 2013). Further, also THAs with femoral head sizes larger than 36 mm (n = 1,205) were excluded due to increased volumetric polyethylene wear (Cooper and Della Valle 2014). Patients with a dual mobility acetabular cup (n = 151), a THA stem combined with a HRA femoral head (n = 30), or with long femoral stem (n = 1) were excluded, as were patients registered as emigrated or dead at date of surgery (n = 66).

### Definitions

Patients entered the study on the date of primary surgery and were followed until revision, death, emigration, or end of study period (October 24, 2016), whichever came first. Revision was defined as a new surgical intervention including partial or complete removal or exchange of the implant. Revision for any reason was considered as primary endpoint and aseptic loosening, dislocation, femoral fracture, and “other” revision causes were considered secondary endpoints. Time since operation was chosen as the underlying timescale in the time-to-event analysis and death was considered a competing risk. Patients with cementless MoP THA were used as reference, as cementless MoP bearings were considered standard.

### Statistics

Patients with HRA and Mitch PER were matched to patients with MoP THA using a propensity score calculated on sex, age (as a continuous variable), year of surgery (as categorical variable), osteoarthritis (OA) as diagnosis, and CCI score as these may influence the outcome (Johnsen et al. 2006, Deleuran et al. 2015, Danish Arthroplasty Registry 2018). We used nearest-neighbor matching with no replacement of controls to simplify the statistical analysis. The balance in baseline variables was examined using standardized differences, where an absolute value below 10% was regarded as balanced (Austin 2014). The number of matched controls for HRA and Mitch PER were determined based on the balance of the baseline variables. We found that 1 THA for every HRA and 5 THAs for every Mitch PER gave a standardized difference below 10% for most variables except for age at surgery (11%) and year of surgery (16%) in the HRA group.

Descriptive statistics were used for the presentation of demographic data and procedure characteristics. The chi-square test was used to compare proportions, and the 2-sample Wilcoxon rank-sum test was used to compare ages and follow-up time because of skewness of these distributions. Median and interquartile range (IQR) are given for age and follow-up time. Cumulative incidence of any revision was computed using the Aalen–Johansen estimator accounting for competing risk. The Aalen–Johansen method estimates the patient’s risk of undergoing a revision as a function of time since operation (Ranstam et al. 2011, Andersen and Keiding 2012, Lacny et al. 2015).

Multivariable regression based on the pseudo-value observation (Klein et al. 2007) was calculated at the pre-specified time-points 2, 4, 6, and 8 years after surgery for Mitch PER and 2, 4, 6, 8, and 10 years for HRA. Once the pseudo-observations had been computed, a model for relative risk (RR) for the uncensored data was applied via generalized estimating equation. In practice, the generalized estimating equation can be obtained in a generalized linear model for the pseudo-observations (Parner and Andersen 2010). We performed stratified analysis on sex, age, OA as diagnosis, comorbidity, and on the different designs for HRA. Stratified analysis was performed at 10 years for HRA and 8 years for Mitch PER.

Any p-value < 0.05 was considered significant and 95% confidence intervals (CI) were calculated. Statistical analyses were carried out using Stata statistical software, release 14.2 (StatCorp, College Station, TX, USA).

### Ethics, funding, and potential conflicts of interest

This study was approved by the Danish Data Protection Agency (journal no. 2008-58-0035). Research was funded by grants from Lillebaelt hospital and the Southern Region of Denmark. No conflict of interest was reported by the authors. 

## Results

### Description of study population (Tables 1 and 2)

29,625 cementless MoP THA, 1,056 HRAs, and 202 Mitch PER with complete information on sex, age, diagnosis, comorbidity, year of surgery, surgical approach, and femoral head size were included in the analyses.

Among patients with HRA, the median follow-up was 8 (IQ 6.4–9.4) years for HRA and 8 (IQR 6.1–9.0) years for matched MoP THA (p = 0.05). The median follow-up was 7 (IQR 6–8) years for Mitch PER and 7 (IQR 6–7) years for the matched MoP THA (p = 0.003).

The median age was 55 (IQR 48–60) years for HRAs and 56 (IQR 50–61) years for Mitch PER. The most common diagnosis was OA, accounting for 86% of HRA and 83% of Mitch PER. HRA was used between 2005 and 2012 and Mitch PER between 2008 and 2011. HRAs were used in 15 different hospitals and Mitch PER in only 1 hospital.

### Risk of any revision of HRA

**Figure 3.**
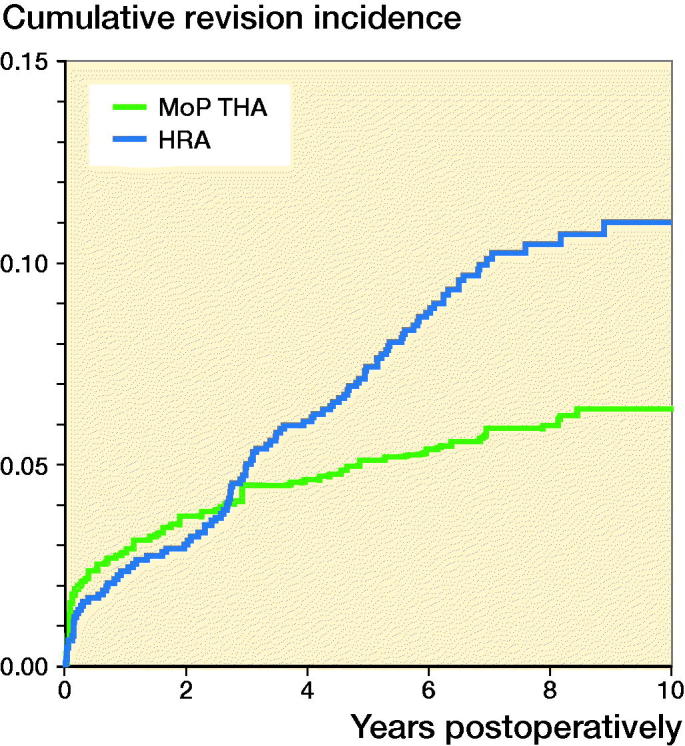
Cumulative incidence for any revision for hip resurfacing arthroplasty (HRA) and propensity score matched cementless metal-on-polyethylene total hip arthroplasty (MoP THA).

At 10 years’ follow-up, the cumulative incidence of any revision of HRA was 11% (9–13) and 6.4% (6–7) of MoP THA (Figure 3). At 4, 6, 8, and 10 years there was a statistically significantly higher RR of any revision of HRA compared with MoP THA (Table 3).

**Table 3.** Relative risk (RR) of any revision with 95% confidence interval (CI) for hip resurfacing arthroplasty (HRA), unmatched cementless metal-on-polyethylene total hip arthroplasty (MoP THA), and propensity-matched MoP THA

Factor	Patients at the start of the period	Relative risk of revision	
		before matching RR (95% CI)	after matching RR (95% CI)
0–2-year follow-up			
HRA	1,056	0.8 (0.6–1.1)	1.0 (0.6–1.6)
MoP THA	1,056	–	1 (ref.)
Unmatched MoP THA	29,625	1 (ref.)	–
2–4-year follow-up			
HRA	1,020	1.5 (1.2–2.0)	1.5 (1.0–2.2)
MoP THA	1,010	–	1 (ref.)
Unmatched MoP THA	27,891	1 (ref.)	–
4–6-year follow-up			
HRA	978	2.4 (1.9–3.2)	1.7 (1.2–2.3)
MoP THA	977	–	1 (ref.)
Unmatched MoP THA	27,085	1 (ref.)	–
6–8-year follow-up			
HRA	944	2.9 (2.1–4.0)	1.6 (1.2–2.2)
MoP THA	942	–	1 (ref.)
Unmatched MoP THA	26,565	1 (ref.)	–
8–10-year follow-up			
HRA	922	2.9 (1.8–4.8)	1.5 (1.1–2.1)
MoP THA	917	–	1 (ref.)
Unmatched THA	26,343	1 (ref.)	–

Most HRA revisions occurred in 2012 and 2013, accounting for 34% (n = 37) and 16% (n = 17), respectively.

### Risk of any revision of Mitch PER

**Figure 4.**
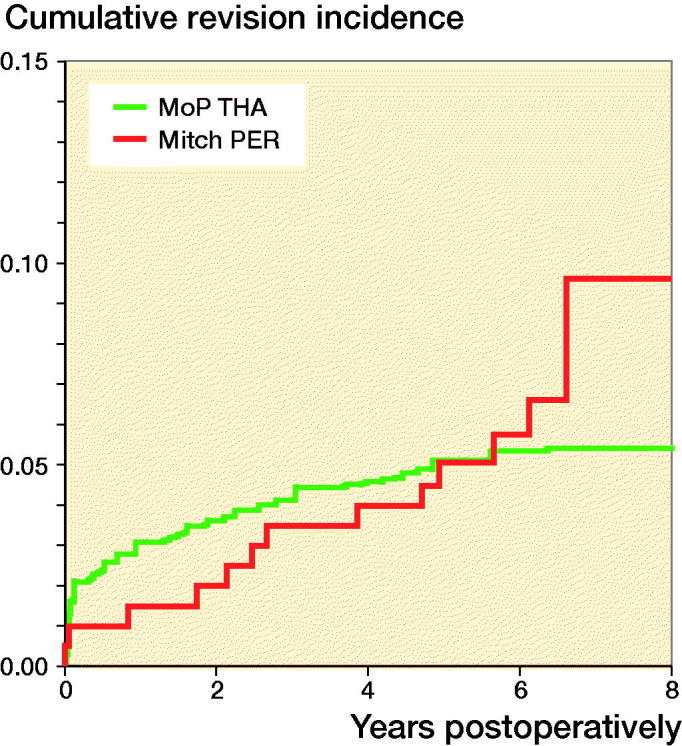
Cumulative incidence for any revision for Mitch proximal epiphyseal replacement (PER) and propensity score matched cementless metal-on-polyethylene total hip arthroplasty (MoP THA).

The cumulative incidence of any revision of the Mitch PER was 10% (4–18%) and 5.4% (5–6%) for MoP THA at 8 years (Figure 4). There was a not statistically significant difference in the RR of any revision for the Mitch PER compared with MoP THA at 8 years (Table 4, see Supplementary data). Among all the revisions of the Mitch PER, 41% (n =5) were in 2013.

### Stratified analysis

HRAs had a statistically significantly higher risk of revision for women (2, CI 1–3), for patients younger than 60 years (2, CI 1–2), for patients diagnosed with OA (2, CI 1–2), and for patients with no comorbidity (2, CI 1–2) compared with THAs at 10 years’ follow-up. There was no significant difference in risk of revision for men, patients aged 60 years or older, patients with other diagnoses than OA, or any comorbidity (CCI score > 0).

**Figure 5.**
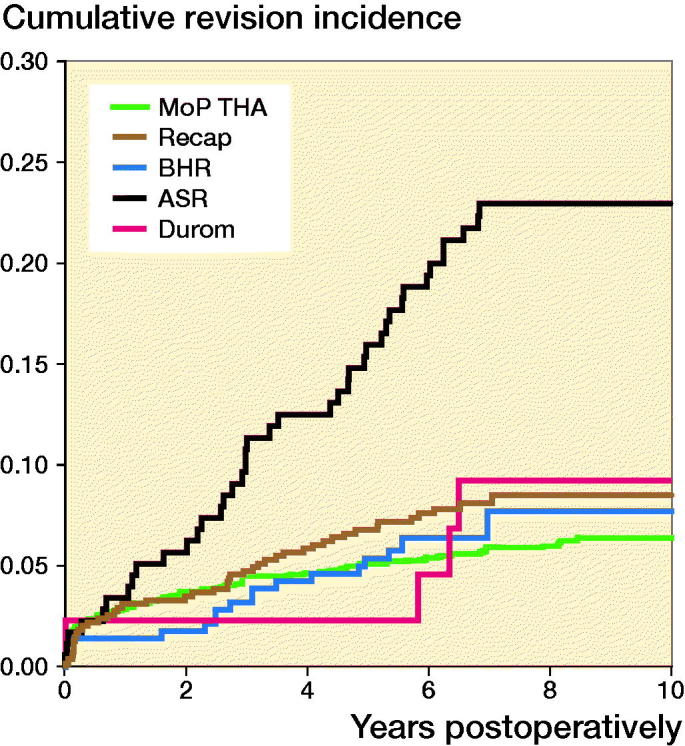
Cumulative incidence for any revision for different designs of resurfacing arthroplasty and propensity score matched cementless metal-on-polyethylene total hip arthroplasty (MoP THA).

For different designs of HRAs at 10 years’ follow-up (Figure 5), the RR of revision for any reason was higher for the ASR component compared with MoP THA (3, CI 2–5) (Table 5, see Supplementary data).

After excluding patients with ASR components, the cumulative incidence of any revision at 10 years’ follow-up was 11 (CI 9–12). The cumulative incidence for ASR alone was 23 (CI 17–29)

The RR of revision for any reason was not statistically significantly different (1, CI 1–2) for the HRA compared with MoP THA at 10 years’ follow-up, when excluding patients with ASR components.

Regarding Mitch PER, there was no statistically significant difference in risk of revision for women, men, patients younger than 60 years, patients older than 60 years, diagnosed with OA, with other diagnoses than OA, and no or any comorbidity compared with MoP THA at 8 years’ follow-up.

### Causes of revision (Table 6, see Supplementary data)

Among HRA revisions, pain, femoral fracture, and “other reasons” dominated, whereas only a few were revised due to dislocation compared with the MoP THAs (p < 0.001).

At 10 years’ follow-up, the RR of revision due to femoral fracture was higher for HRA than MoP THA (3, CI 1–9) but the RR of revision due to dislocation (0, CI 0–0.5) was lower. We found no statistically significant difference in RR of revision due to aseptic loosening.

The majority of Mitch PER were revised due to femoral fracture. At 8-year follow-up, the RR of revision due to fracture was markedly higher for Mitch PER than MoP THA (34, CI 4–279) and the RR of revision due to dislocation (0, CI 0–0.4) and “other causes” (0, CI 0–0) was lower. We found no statistically significant difference in RR of revision due to aseptic loosening.

## Discussion

In this nationwide, register-based study from DHR, we found higher overall revision of HRA compared with MoP THA. However, if patients with ASR were excluded from the study population the RR of revision for any reason was similar for HRA and uncemented MoP THA. The risk of revision of Mitch PER was not statistical significantly different compared with that of MoP THA after 8 years. Most of the revisions were performed in the early period after the Danish national guidelines advocated discontinuing the use of MoM bearings.

### Revision for any reason

In the Australian Orthopaedic Association National Joint Replacement Registry (AOANJRR) (Australian Orthopaedic Association 2017), the cumulative incidence of revision for any reason was 9.6 (CI 9.1–10) for HRA after 10 years’ follow-up, which is lower than in our study. This could be explained by differences in the use of specific component designs. In our study, 16% of the HRAs were ASR while in Australia only 7% of the HRAs were ASR. In the National Joint Registry for England, Wales, Northern Ireland and the Isle of Man (NJR) (National Joint Registry 2017), the cumulative percentage probability of revision was 11.2 (10.8–11.5). In the NJR, Kaplan–Meier estimates were used, which could account for the increased risk of revision, since the Kaplan–Meier estimator may overestimate the risk of revision by not accounting for death as a competing risk (Gillam et al. 2010). The Mitch PER had a lower cumulative incidence of revision at 8 years than the 8-year cumulative incidence for the HRAs in our study (10%; CI 8.5–12%), which could indicate that Mitch PER might have a survival rate similar to some of the HRAs.

### Causes of revision

We found that the most common cause of revision for HRA was “other causes” and pain, followed by loosening and fracture of the femoral neck, which are not comparable to the AOANJRR (where it was aseptic loosening, metal-related pathology, and fracture of the femoral neck) or to the NJR (where pain, metal-related pathology, and aseptic loosening were the most common causes).

The reason for this could be that revisions due to “metal-related pathology” such as ARMD have not been registered in the DHR and would likely be classified as “other causes” or “pain.”

The Mitch PER was designed to protect bone of the proximal femur, to minimize the risk of femoral neck fracture. However, the most common cause of revision of the Mitch PER was fracture of the femoral neck followed by aseptic loosening. This could indicate that the Mitch PER may not have the femoral bone protecting abilities as previously believed.

### Strengths and limitations

The strengths of our study include the population-based design with prospectively collected data, the large sample size, and the complete follow-up that limits possible selection bias. Further, the DHR is a medical database with independently registered data with moderate to high validity (Danish Hip Arthroplasty Registry 2018). Although the DHR has been validated, prosthetic joint infection is the only revision cause that has been validated (Gundtoft et al. 2016). Hence, misclassifications of revisions cannot be fully accounted for.

There are several limitations that should be considered when interpreting the results. We excluded 433 THAs with missing data on surgical approach (n = 367), or death or emigration (n = 66). However, we assume that they would have no influence on the results in this large study population. Even though the use of cumulative competing risk analysis and the regression with the pseudo-value approach is based on assumption of independent observations, we chose not to exclude bilateral hips because a previous published guideline reported that the bilateral issue in register settings has little practical consequence when the outcome studied is revision (Ranstam et al. 2011).

Even though we used propensity score matching to account for several confounders, there is still the possibility of unmeasured confounding. We did not include femoral head size in the propensity score matching even though it is a well-documented risk factor (Smith et al. 2012), as THA has smaller femoral head size than HRA and thereby could be considered a proxy for the different groups in this study. Further, the DHR does not contain any information on potential confounders such as blood concentrations of chromium and cobalt ion levels, height, weight, BMI, physical activity before and after surgery, or medication before and after surgery.

We used nearest-neighbor matching to match HRAs to MoP THA but could not meet our criteria of a standardized difference below 10% for year of surgery (19%). However, this should not have any impact on the results, as the difference is small and the surgical technique for THA surgery has not been significantly altered for several years.

In our stratified analysis we divided the HRAs and Mitch PER into smaller groups. This might leave room for a type II error due to the small numbers in each group, especially the Mitch PER group.

The increased risk of revision for any reason found among the HRAs might be influenced by the tendency to revise HRAs earlier because of the increased focus on ARMD and pseudotumors since January 2012 when a documentary concerning the dangers of MoM bearings was released in Denmark.

This might be the reason for the increased number of revisions seen in 2012 and 2013 for the HRAs and Mitch PER and could shorten the survival compared with THAs. Surgeons may have been more prone to do revision surgery due to pressure from different stakeholders including orthopedic surgeons, administrative systems, patients, press, and industry. Or the increased risk of revision could be real and have no relation to the increased focus.

All surgeries with the Mitch PER were performed at the same hospital and by one and the same surgeon. This could influence the results depending on the surgeon’s preferences (confounding by indication) and threshold for revision. While the RR of revision for any reason at 8 years was not significant, the rising RR might indicate that the risk of revision for any reason may become significant with longer follow-up.

### Conclusion

We showed that the HRA had an increased risk of revision compared with the MoP THA at 10 years’ follow-up. When excluding ASR, the HRA group had a similar risk of revision compared with MoP THA.

Mitch PER did not have a statistically significantly increased risk of revision, but as the RR is increasing every 2nd year together with the broad confidence interval, this might indicate that with longer follow-up the results could have shown a statistically significantly increased revision risk.

The most common cause of revision of the Mitch PER was femoral fracture. Hence, this prosthesis does not protect the femur from fracture as computer simulation has previously indicated. We found revisions of HRA during the whole follow-up even 10 years after implantation, which is why we suggest that these patients should be followed clinically.

### Supplementary data

Tables 4–6 are available as supplementary data in the online version of this article, http://dx.doi.org/10.1080/17453674. 2019.1646201

**Table 1.** Patient- and surgery-related characteristics for the patients who received hip resurfacing arthroplasty (HRA) or cementless metal-on-polyethylene total hip arthroplasty (MoP THA)

Factor	HRA n = 1,056	MoP THA (matched) n = 1,056	MoP THA (full cohort) n = 29,625	Stand. diff. **^a^**	p-value
Sex				0.05	0.2
Female	287 (27)	290 (28)	16,096 (54)		
Male	769 (73)	766 (73)	13,529 (46)		
Age at operation				0.10	< 0.001
< 49	332 (31)	285 (27)	1,939 (7)		
50–59	463 (44)	360 (34)	4,432 (15)		
60–69	248 (24)	345 (33)	11,724 (40)		
70–79	13 (1)	61 (6)	9,278 (31)		
> 80	0 (0)	5 (1)	2,252 (8)		
Diagnosis				–0.10	< 0.001
Primary OA	907 (86)	867 (82)	25,236 (85)		
Trauma	12 (1)	46 (4)	1,742 (6)		
Femoral head osteonecrosis	3 (0)	24 (2)	718 (2)		
Arthritis	6 (1)	10 (1)	299 (1)		
Childhood hip disorders	120 (11)	88 (8)	1,372 (5)		
Other	8 (1)	21 (2)	258 (1)		
Year of surgery				0.20	0.01
2005	24 (2)	20 (2)	26 (0)		
2006	213 (20)	141 (13)	223 (1)		
2007	190 (18)	206 (20	665 (2)		
2008	164 (16)	180 (17)	1,125 (4)		
2009	218 (21)	221 (21)	2,010 (7)		
2010	168 (16)	201 (19)	3,054 (10)		
2011	66 (6)	70 (7)	3,792 (13)		
2012	13 (1)	17 (2)	4536 (15)		
Charlson comorbidity index at surgery				0.06	0.4
Low	904 (86)	881 (83)	23,543 (80)		
Medium	120 (11)	137 (13)	4414 (15)		
High	32 (3)	38 (4)	1668 (6)		

Values are numbers of patients and percentages (%) within each group.

a Standardized difference and p-value are between HRA and matched MoP THA.

**Table 2.** Patient- and surgery-related characteristics for the patients who received the Mitch proximal epiphyseal replacement (PER) or cementless metal-on-polyethylene total hip arthroplasty (MoP THA)

Factor	Mitch PER n = 202	MoP THA (matched)n = 1,010	MoP THA (full cohort) n = 29,625	Stand. diff. **^a^**	p-value
Sex				0.06	0.4
Female	50 (25)	223 (22)	16,096 (54)		
Male	152 (75)	787 (78)	13,529 (46)		
Age at operation				0.09	< 0.001
< 49	51 (25)	267 (26)	1,939 (7)		
50–59	96 (48)	291 (29)	4,432 (15)		
60–69	49 (24)	373 (37)	1,1724 (40		
70–79	5 (3)	77 (8)	9,278 (31)		
> 80	1 (1)	2 (0)	2,252 (8)		
Diagnosis				0.03	0.1
Primary OA	168 (83)	858 (84)	25,236 (85)		
Trauma	7 (4)	31 (3)	1,742 (6)		
Femoral head osteonecrosis	13 (6)	28 (3)	718 (2)		
Arthritis	1 (1)	9 (1)	299 (1)		
Childhood hip disorders	12 (6)	73 (7)	1,372 (5)		
Other	1 (1)	16 (2)	258 (1)		
Year of surgery:				0.05	0.9
2008	23 (11)	117 (11)	1,125 (4)		
2009	77 (38)	374 (38)	2,010 (7)		
2010	67 (33)	326 (34)	3,054 (10)		
2011	35 (17)	193 (17)	3,792 (13)		
Charlson comorbidity index at surgery				–0.02	1.0
Low	180 (90)	908 (90)	23,543 (80)		
Medium	19 (9)	88 (9)	4,414 (15)		
High	3 (2)	14 (1)	1,668 (6)		

Values are numbers of patients and percentages (%) within each group.

a Standardized difference and p-value are between Mitch PER and matched MoP THA.

## Supplementary Material

Supplemental Material
