# Supplementary material for: Survival and revision causes of hip resurfacing arthroplasty and the Mitch proximal epiphyseal replacement: results from the Danish Hip Arthroplasty Register
Source: Acta Orthop. 2019 Jul 25;90(6):523–9. doi: 10.1080/17453674.2019.1646201 (PMC6844433; doi:10.1080/17453674.2019.1646201)
Supplement: Supplemental Material [file IORT_A_1646201_SM2582.pdf]

Supplementary data

Table 4. Relative risk (RR) of any revision with 95% confidence interval (CI) for Mitch proximal epiphyseal replacement (PER), unmatched cementless metal-on-polyethylene total hip arthroplasty (MoP THA), and propensity-matched MoP THA

Factor	Patients at the start of the period	Relative risk of revision before matching RR (95% CI)	Relative risk of revision after matching RR (95% CI)
0–2-year follow-up			
Mitch PER	202	0.4 (0.1–1.4)	0.5 (0.2–1.7)
MoP THA	1,010	–	1 (ref.)
Unmatched MoP THA	29,625	1 (ref.)	–
2–4-year follow-up			
Mitch PER	195	0.9 (0.4–2.0)	0.9 (0.4–2.0)
MoP THA	972	–	1 (ref.)
Unmatched MoP THA	27,891	1 (ref.)	–
4–6-year follow-up			
Mitch PER	188	1.4 (0.6–3.3)	1.1 (0.6–2.2)
MoP THA	940	–	1 (ref.)
Unmatched MoP THA	27,085	1 (ref.)	–
6–8-year follow-up			
Mitch PER	181	3.1 (1.3–7.5)	2.0 (0.9–4.3)
MoP THA	919	–	1 (ref.)
Unmatched MoP THA	26,565	1 (ref.)	–

Table 5. Stratified analysis with relative risk (RR) of any revision with 95% confidence intervals (CI) at 10 years' follow-up among different designs of hip resurfacing arthroplasty (HRA) compared with matched cementless metal-on-polyethylene total hip arthroplasty (MoP THA)

Type	n (%)	Follow-up time median (IQR)	Risk of revision RR (95% CI)
Recap	548 (52)	7.7 (6.4–9.2)	1.2 (0.8–1.7)
BHR	286 (27)	7.0 (6.1–8.9)	1.1 (0.6–1.8)
ASR	177 (17)	8.6 (6.7–10.0)	3.2 (2.2–4.7)
Durom	45 (4)	10.1 (9.6–10.6)	1.8 (0.5–6.7)
Total THA	1,056	7.7 (6.4–9.4)	1 (ref.)

Percentage of total HRA (%) is shown.

Table 6. Main indications for revision of hip resurfacing arthroplasty (HRA), Mitch PER, and their propensity score matched cementless metal-on-polyethylene total hip arthroplasty (MoP THA)

Revision causes	HRA n = 107	MoP THA n = 68	p-value	Mitch PER n = 13	MoP THA n = 44	p-value
Aseptic loosening	18 (2)	18 (2)	1.000	4 (2)	14 (1)	0.5
Component failure	4 (0)	7 (1)	0.4	0 (0)	4 (0)	0.4
Prosthetic joint infection	4 (0)	9 (1)	0.2	0 (0)	10 (1)	0.2
Dislocation	1 (0)	18 (2)	< 0.001	0 (0)	7 (1)	0.2
Femoral fracture	1 (2)	5 (1)	0.004	6 (3)	5 (1)	< 0.001
Other	34 (3)	6 (1)	< 0.001	2 (1)	2 (0)	0.2
Pain	29 (3)	2 (0)	< 0.001	1 (1)	2 (0)	0.1

Total number of revisions and percentage of total HRA or MoP THA are given.
